# Quercetin suppresses cellular migration and invasion in human head and neck squamous cell carcinoma (HNSCC)

**DOI:** 10.7603/s40681-016-0015-3

**Published:** 2016-08-12

**Authors:** Chien-Yi Chan, Chia-Hsien Lien, Ming-Fen Lee, Chun-Yin Huang

**Affiliations:** 1 Department of Nutrition, China Medical University, No. 91 Hsueh-Shih Road, 404 Taichung, Taiwan; 2 Department of Nutrition and Health Sciences, Chang Jung Christian University, 711 Tainan, Taiwan

**Keywords:** HNSCC, Quercetin

## Abstract

Head and neck squamous cell carcinoma (HNSCC) with aberrant epidermal growth factor receptor (EGFR) signaling is often associated with a poor prognosis and a low survival rate. Hence, efficient inhibition of the EGFR signaling-mediated malignancy would improve survival rate. In a previous study, we demonstrated that quercetin appears to be a potent anti-tumorigenic agent through its inhibition of the EGFR/Akt pathway in oral cancer, but its anti-metastatic potential in HNSCC remains unclear [1]. Here, we have hypothesized that quercetin might be effective in metastatic inhibition in EGFR-overexpressing HNSCC cells. Quercetin treatment with 10 μM (half concentration of IC50) suppressed cell migration and invasion in EGFR-overexpressing HSC-3 and FaDu HNSCC cells. Quercetin also inhibited the colony growth of HSC-3 cells embedded in a Matrigel matrix. Among matrix metalloproteinases (MMPs), the secreted gelatinases MMP-2 and MMP-9 are responsible for the degradation of gelatin in the extracellular matrix and type IV collagen in the basement membrane; and this degradation event is crucial for the migration from the origin and the invasion into the bone in HNSCC. Quercetin (10 μM) treatment also suppressed the expression and proteolytic activity of MMP-2 and MMP-9. Taken together, our data indicate that quercetin is an effective anti-cancer agent against MMP-2- and MMP-9-mediated metastasis in EGFR-overexpressing HNSCC.

## 1. Introduction

Epidermal growth factor (EGF) receptor (EGFR) gene is frequently amplified in head and neck squamous cell carcinoma (HNSCC) [2]. Increased expression of EGFR and its ligand are often associated with poor clinical outcome—that is, high recurrence and low survival rates [3-5]. EGFR activation is often implicated in malignant phenotypes of cancer cells: increased survival, antiapoptosis, angiogenesis, and metastatic potential through signal transduction pathways, such as phosphoinositide 3-kinase (PI3K)/Akt and RAS/extracellular signal-regulated kinases (ERK)[6, 7]. Matrix metalloproteinase(MMP) family are involved in the integrity regulation of the extracellular microenvironment [8]. Inhibition of the PI3K/Akt signal transduction pathway may result in the reduction of MMP proteins, indicating an important role of the EGFR/Akt/MMP signal axis in controlling the metastatic potential of cancers.

MMP family members, including MMP-2 and MMP-9, play a crucial role in cancer malignancy and metastasis [9-11]. Higher expression levels of MMP-2 and MMP-9 have been found in oral cancer cells when compared to their normal mucosal counterparts [12-14]. Clinically, drugs available for oral cancer patients are mainly designed to target EGFR [15-17]; however, mutations of the EGFR downstream effectors and resistance to said drugs have been reported in some patients [5]. Therefore, to improve therapeutic efficacy, new anti-cancer agents should be considered accordingly.

Quercetin (3, 3’, 4’, 5, 7-pentahydroxyflavone), a major dietary flavonol, exists naturally in a wide range of fruits, vegetables, and their products, including onions, apples, and red wine [18]. In addition to its antioxidant, anti-inflammatory, and antiproliferative and proapoptotic properties [18], quercetin has also been widely investigated for its potential to inhibit both cellular migration and the invasion of cancer cells, including glioblastoma [19, 20], melanoma [21], prostate cancer [22], breast cancer [23, 24], and oral cancer cells [25, 26]. Mechanistically, quercetin may inhibit cellular migration and invasion through the deactivation of MMP-2 and/or MMP-9 [20, 23, 24, 26]; however, evidence regarding the anti-metastatic efficacy of quercetin in invasive HNSCC is still limited. Therefore, the current study is aimed at investigating the effects of quercetin on the invasiveness of aggressive HNSCC cells.

In a previous study, we demonstrated that quercetin is a potent anti-growth agent in EGFR-overexpressing oral cancer cells, where quercetin inhibits cell growth and induces apoptosis through modulation of the EGFR/Akt/FOXO1 axis [1]. In this current study, we investigated the inhibitory effect of quercetin on cellular migration and invasion in human HNSCC cell lines with lymph node metastasis [27, 28], and we further identified the suppressive role of quercetinin in MMP-2 and MMP-9-mediated invasion.

## 2. Materials and methods

### 2.1. Reagents and antibodies

All chemicals including quercetin were purchased from Sigma (St. Louis, MO) unless specified otherwise. Antibodies for MMP-2 and MMP-9 were purchased from Abcam (Burlingame, CA). Polyvinylidenedifluoride (PVDF) membranes and enhanced chemiluminescence (ECL) detection reagents were bought from Perkin Elmer Life Sciences, Inc. (Waltham, MA).

### 2.2. Cell culture and treatment

HSC-3 and FaDu human HNSCC cells were kindly gifted to us by Drs. Tzong-Ming Shieh and Tzong-Der Way at China Medical University (Taichung, Taiwan), respectively. FaDu and HSC-3 cells were maintained in Dulbecco’s Modified Eagle Medium (DMEM) and DMEM-F12 (Invitrogen), respectively, supplemented with 10% fetal bovine serum (FBS) and 1% antibioticantibimycotic (Gibco). Cells were maintained in an incubator with 5% CO_2_ at 37°C.

### 2.3. Western blot analysis [1]

Cells were washed with cold PBS and lysed in a RIPA buffer containing 150 mM NaCl, 10 mM Tris (pH 7.2), 0.1% sodium dodecyl sulfate, 1% Triton X-100, 1% deoxycholate, 5 mM EDTA, and protease/phosphatase inhibitors. Protein concentration was determined by BCA protein assay, and denatured proteins were separated in 10% sodium dodecyl sulfate-polyacrylamide gels (SDS-PAGE) and transferred onto PVDF membranes. Nonspecific binding was blocked with 5% milk in a TBST buffer (20 mM Tris base, 140 mM NaCl, pH 7.6, 0.1% Tween-20) for 1 h, followed by incubation with primary antibodies at 4°C overnight and secondary antibodies at room temperature for 1 h. Blots were visualized by ECL detection reagents.

### 2.4. Wound-healing assay

HSC-3 and FaDu cells (3 × 10^4^ cells) were seeded onto a Cultureinsert 2 well (ibidi, Munich, Germany) which was placed onto a 12-well plate. After 24 h of attachment, the Culture-insert 2 well was removed and cells were incubated in a medium containing various concentration of quercetin (0-10 μM). Wound healing was observed and photographed every 2 hours under a microscope with 200× magnification.

### 2.5. Matrigel invasion assay [29]

**Figure 1:**
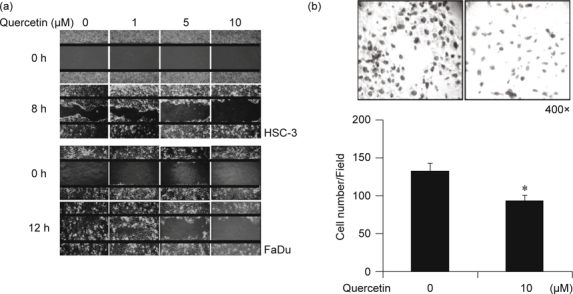

Matrigel inserts for 24-well chambers were obtained from BD Biosciences (Bedford, MA) and used according to the manufacturer’s protocol. HSC-3 cell suspensions (3 × 10^4^ cells) were added to the upper chamber, with or without 10 μM of quercetin in a serum-free growth medium, and a chemoattractant (10% FBS-containing medium) was added to the lower chamber. After 48 h of incubation in a 370C, 5% CO2 incubator, the non-invading cells from the upper chamber were removed using cotton swabs, and the cells on the lower surface were fixed with 100% methanol, stained (Giesma in 20% ethanol), and counted. Cell invasion was photographed under 400× magnification. The invaded cells were counted in five randomly selected microscopic fields (200× magnification). Error bars in Fig. 1B represent the variation of the cell numbers between the selected fields.

### 2.6. Gelatin zymography [29]

HSC-3 and FaDu cells (1 × 10^5^ cells) were incubated in a growth medium supplemented with quercetin for 24 h. The media were then collected and separated on 8% SDS-PAGE containing 0.1% (w/v) gelatin. After separation, the gels were washed in a renaturing buffer containing 2.5% Triton X-100 at room temperature for 30 min and then incubated in a developing buffer containing 50 mM Tris-HCl (pH 7.5), 200 mM NaCl, 5 mM CaCl2, and 0.02% Brij 35 at 37°C for 16 h. Lastly, the gels were stained with 0.2% Coomassie blue and distained in 50% methanol, 10% acetic acid, and 40% water. The proteolytic activity of the indicated MMPs was detected as a clear band against a dark blue background.

### 2.7. Colony formation in a 3D Matrigel model

A Lab-Tek® II Chamber slide (Thermo Fisher Scientific, Waltham, MA) was pre-coated with a 70 μl Matrigel matrix and polymerization was allowed for in a 37°C incubator for 10 to 15 minutes. Cells in the growth medium mixed with the indicated concentration 0 or 10 μM of quercetin and 2% Matrigel matrix were transferred onto the chamber slide. Once the gel was polymerized, cells were allowed to grow with the changing media every other day for 6 days. Colony formation was photographed under 200× magnification.

### 2.8. Quantitativereal-time polymerase chain reaction (qPCR)

Cellular RNA was extracted using an RNeasy Mini Kit (Qiagen, Valencia, CA) according to the manufacturer’s instructions, and reverse-transcribed into cDNA using the iScript cDNA Kit (Bio- Rad,Hercules, CA) again according to the manufacturer’s instructions. Then, quantitative real time PCR was carried out using the Bio-Rad iQ SYBR Green Supermix (Bio-Rad, Hercules, CA) with the MJ Mini^TM^ Thermal Cycler equipped with Bio-Rad CFX Manager software (Bio-Rad, Hercules, CA) [30]. The following primers were used: human MMP-2, 5’-CATCAAGTTCCCCGGCGATG- 3’(F) and 5’-AAACAGGTTGCAGCTCTCCT-3’(R); MMP-9,5’-CTTTTGAGTCCGGTGGACGAT-3’(F) and 5’- TCGCCAGTACTTCCCATCCT-3’(R); 18S, 5’-GTCTGTGATGCCCTTAGATG- 3’(F) and 5’-AGCTTATGACCCGCACTTAC- 3/(R).

### 2.9. Statistical analysis

Data are expressed as mean ± SD from at least three independent experiments. Statistical significance was analyzed using Student’s *t* test. Results were considered significantly different at *p* < 0.05.

## 3. Results

### 3.1. Quercetin inhibits cell migration, invasion, and colony growth in human HNSCC cells

**Figure 2:**
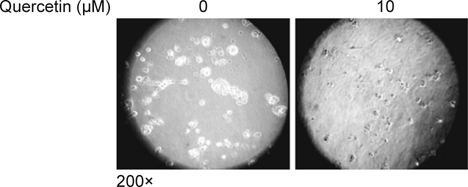

Again, in a previous study, we demonstrated that EGFR-overexpressing oral cancer cells are sensitive to the growth-inhibitory effect of quercetin [1]. However, its anti-metastatic efficacy potential regarding EGFR-overexpressing HNSCC cells remains elusive and unclear. In the present study, we exposed two human HNSCC cell lines, HSC-3 and FaDu, to various concentrations (0 to 10 μM) of quercetin, quantities which are all much less than the IC50 (20 μM) of quercetin for HSC-3 cells [1]. Quercetin dose-dependently inhibited cellular migration in both HSC-3 and FaDu cells as evidenced by delayed wound-healing time (Fig. 1A). Further, administration of 10 μM of quercetin also significantly suppressed the ability of HSC-3 cells to invade through a Matrigel basement membrane (Fig. 1B). These results indicate that the metastatic HSC-3 and FaDu cell lines are in fact susceptible to quercetin. Indeed, quercetin supplementation for 6 days significantly suppressed HSC-3 colony formation in a 3D Matrigel model that mimics the physiological cell-cell and cellextracellular matrix (ECM) interaction (Fig. 2). Taken together, these data support the possibility of quercetin as a potential antimetastatic reagent in human HNSCC.

### 3.2. Quercetin modulates MMP-2 and MMP-9 activation

**Figure 3:**
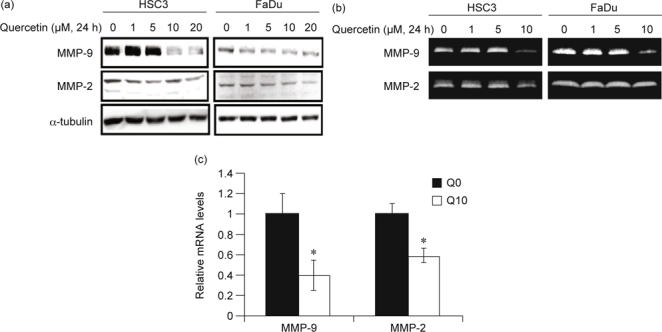

MMP-2 and MMP-9 are secreted gelatinases responsible for the degradation of gelatin in the ECM and type IV collagen in the basement membrane, and both are also implicated in the migration from the origin and the invasion into the bone in HNSCC [27]. With this in mind, we examined the effect of quercetin on the activation of MMP-2 and MMP-9. After 24 h of treatment, quercetin displayed inhibitory efficacy on the protein levels of both MMP-2 and MMP-9 (Fig. 3A). Low concentrations of quercetin (1-5 μM) had almost no effect on MMP-9 protein levels while high concentrations of quercetin (10-20 μM) potently suppressed MMP-9 protein levels; however, quercetin only mildly suppressed MMP-2 protein levels in a dose-dependent manner (Fig. 3A). The effect of quercetin on the proteolytic activity of MMP-9 and MMP-2 was in agreement with that of the protein expression level (Fig. 3B). Finally, we examined whether or not the observed reduction of MMP protein levels was due to decreased transcription. The qPCR data confirmed that the mRNA expression levels of both MMP-9 and MMP-2 were significantly reduced upon receiving 10 μM of quercetin treatment (Fig. 3C).

Taken as a whole, our results indicate that quercetin at a concentration of 10 μM potently inhibits cellular migration and invasion, at least in part, through the modulation of MMP-9 and MMP-2 activation in EGFR-overexpressing HNSCC.

## 4. Discussion

The deregulated EGFR signaling pathway associated with cancer malignancy is often found in HNSCC [31]. Again, we have demonstrated in a previous study that quercetin is a potent inhibitor of the EGFR/PI3K/Akt pathway-mediated cell growth in the EGFR-overexpressing HSC-3 oral cancer cell line [1]. In the current study, we further identified that quercetin at a concentration of 10 μM also displays anti-metastatic potential as evidenced by the suppression of cellular migration, invasion, and colony formation in a 3D Matrigel model in EGFR-overexpressing HNSCC cells (Figs. 1-2). Our data suggest that HNSCC with EGFR overexpression seems quite sensitive to the anti-cancer efficacy of quercetin.

Aberrant EGFR signaling activation is correlated with the expression of MMPs including MMP-2 and MMP-9 [32]. MMP-2 and MMP-9 are highly implicated in cellular growth, migration, and invasion of HNSCC [32]; MMP-2 is significantly correlated with metastasis to lymph nodes while MMP-9 is involved in the control of tumor neovascularization [32]. Although MMP-9 is not highly expressed in HSC-3 cells [27], its expression is potently induced upon EGF stimulation and is responsible for EGF-mediated cellular invasion [33]. Thus, reagents targeting MMP-2 and MMP-9 may help suppress HNSCC metastasis. Here, we have demonstrated that quercetin (10 μM) significantly suppressed the transcriptional activation of MMP-2 and MMP-9 in both HSC-3 and FaDu cells (Fig. 3). These data indicate that quercetin may inhibit metastasis of EGFR-overexpressing HNSCC through the down-regulation of MMP-2 and MMP-9.

In summary, our data support the possibility of quercetin as an efficient anti-cancer agent in EGFR-overexpressing HNSCC. Quercetin efficiently inhibits the cellular migration and invasion of the HNSCC cell lines, HSC-3 and FaDu. In addition, quercetin also inhibits the colony formation of HSC-3 cells surrounded with Matrigel matrix. Our data show that the activation of gelatinase MMP-2 and MMP-9 was suppressed by quercetin administration. As illustrated in Fig. 4, quercetin may inhibit migration and invasion of the HNSCC cells, in part, through suppressing the expression of MMP-2 and MMP-9. Collectively, our data indicate quercetin is a potential alternative regimen for HNSCC patients carrying an aberrant EGFR signaling axis.
